# Large-scale evaluation of outcomes after a genetic diagnosis in children with severe developmental disorders

**DOI:** 10.1016/j.gimo.2024.101864

**Published:** 2024-10-14

**Authors:** Harriet Copeland, Karen J. Low, Sarah L. Wynn, Ayesha Ahmed, Victoria Arthur, Meena Balasubramanian, Katya Bennett, Jonathan Berg, Marta Bertoli, Lisa Bryson, Catrin Bucknall, Jamie Campbell, Kate Chandler, Jaynee Chauhan, Amy Clarkson, Rachel Coles, Hector Conti, Philandra Costello, Tessa Coupar, Amy Craig, John Dean, Amy Dillon, Abhijit Dixit, Kathryn Drew, Jacqueline Eason, Francesca Forzano, Nicola Foulds, Alice Gardham, Neeti Ghali, Andrew Green, William Hanna, Rachel Harrison, Mairead Hegarty, Jenny Higgs, Muriel Holder, Rachel Irving, Vani Jain, Katie Johnson, Rachel Jolley, Wendy D. Jones, Gabriela Jones, Shelagh Joss, Ruta Kalinauskiene, Farah Kanani, Karl Kavanagh, Mahmudur Khan, Naz Khan, Emma Kivuva, Nayana Lahiri, Neeta Lakhani, Anne Lampe, Sally Ann Lynch, Sahar Mansour, Alice Marsden, Hannah Massey, Shane McKee, Shehla Mohammed, Swati Naik, Mithushanaa Nesarajah, Ruth Newbury-Ecob, Fiona Osborne, Michael J. Parker, Jenny Patterson, Caroline Pottinger, Matina Prapa, Katrina Prescott, Shauna Quinn, Jessica A. Radley, Sarah Robart, Alison Ross, Giulia Rosti, Francis H. Sansbury, Ajoy Sarkar, Claire Searle, Nora Shannon, Debbie Shears, Sarah Smithson, Helen Stewart, Mohnish Suri, Shereen Tadros, Rachel Theobald, Rhian Thomas, Olga Tsoulaki, Pradeep Vasudevan, Maribel Verdesoto Rodriguez, Emma Vittery, Sinead Whyte, Emily Woods, Thomas Wright, David Zocche, Helen V. Firth, Caroline F. Wright, the DDD Study

**Affiliations:** 1 Peninsula Clinical Genetics, Clinical Genetics, Royal Devon University Healthcare NHS Foundation Trust, Exeter, United Kingdom; 2 Bristol Regional Clinical Genetics Service, Level B, St Michael’s Hospital, Bristol, United Kingdom; 3 Centre for Academic Child Health, Bristol Medical School, University of Bristol, Bristol, United Kingdom; 4 Unique (Rare Chromosome Disorder Support Group), Oxted, Surrey, United Kingdom; 5 All Wales Medical Genomics Service, Wales Genomic Health Centre, Cardiff Edge Business Park, Whitchurch, Cardiff, United Kingdom; 6 Manchester Centre for Genomic Medicine, St Mary’s Hospital, Manchester, United Kingdom; 7 Sheffield Clinical Genomics Service, Sheffield Children’s NHS Foundation Trust, Sheffield, United Kingdom; 8 Liverpool Centre for Genomic Medicine, Liverpool Women’s Hospital, Liverpool, United Kingdom; 9 Clinical Genetics, Human Genetics Unit, Ninewells Hospital, Dundee, United Kingdom; 10 Northern Genetics Service, The Newcastle upon Tyne Hospitals NHS Foundation Trust, Institute of Genetic Medicine, International Centre for Life, Newcastle upon Tyne, United Kingdom; 11 West of Scotland Centre for Genomic Medicine, Laboratory Medicine Building, Queen Elizabeth University Hospital, Glasgow, United Kingdom; 12 North of Scotland Regional Genetics Service, Clinical Genetics Centre, Ashgrove House, Aberdeen Royal Infirmary, Foresterhill, Aberdeen, United Kingdom; 13 Yorkshire Regional Genetics Service, Chapel Allerton Hospital, Leeds, United Kingdom; 14 North West Thames Regional Genetics Service, London North West University Healthcare NHS Trust, Northwick Park Hospital, Harrow, United Kingdom; 15 Wessex Clinical Genetics Service, Princess Anne Hospital, Southampton, United Kingdom; 16 South West Thames Centre for Genomics, St. George's University Hospital, Tooting, London, United Kingdom; 17 Nottingham Regional Genetics Service, Nottingham City Hospital Campus, The Gables, Nottingham, United Kingdom; 18 West Midlands Regional Genetics Service, Department of Clinical Genetics, Birmingham Women’s Hospital, Edgbaston, United Kingdom; 19 Department of Clinical Genetics, Guy's Hospital, London, United Kingdom; 20 Department of Clinical Genetics, Children’s Health Ireland, Crumlin, Ireland; 21 Northern Ireland Regional Genetics Service, Medical Genetics Department, Belfast City Hospital, Belfast, United Kingdom; 22 North East Thames Regional Genetics Service, Clinical Genetics Unit, Great Ormond Street Hospital NHS Trust, London, United Kingdom; 23 Leicestershire, Northamptonshire and Rutland Genomic Medicine Service, Leicester Royal Infirmary, University Hospitals of Leicester NHS Trust, Leicester, United Kingdom; 24 South East Scotland Clinical Genetics Service, Western General Hospital, Edinburgh, United Kingdom; 25 East Anglian Medical Genetics Service, Clinical Genetics, Addenbrooke’s Treatment Centre, Addenbrooke’s Hospital, Cambridge, United Kingdom; 26 Oxford Centre for Genomic Medicine, Department of Clinical Genetics, Churchill Hospital, Headington, Oxford, United Kingdom; 27 Department of Clinical and Biomedical Sciences, Medical School, University of Exeter, St Luke’s Campus, Exeter, United Kingdom; 28 Wellcome Sanger Institute, Wellcome Genome Campus, Hinxton, Cambridge, United Kingdom

**Keywords:** Clinical audit, Developmental disorders, Diagnosis, Genomic medicine, Treatment

## Abstract

**Purpose:**

We sought to evaluate outcomes for clinical management after a genetic diagnosis from the Deciphering Developmental Disorders study.

**Methods:**

Individuals in the Deciphering Developmental Disorders study who had a pathogenic/likely pathogenic genotype in the DECIPHER database were selected for inclusion (*n* = 5010). Clinical notes from regional clinical genetics services notes were reviewed to assess predefined clinical outcomes relating to interventions, prenatal choices, and information provision.

**Results:**

Outcomes were recorded for 4237 diagnosed probands (85% of those eligible) from all 24 recruiting centers across the United Kingdom and Ireland. Clinical management was reported to have changed in 28% of affected individuals. Where individual-level interventions were recorded, additional diagnostic or screening tests were started in 903 (21%) probands through referral to a range of different clinical specialties, and stopped or avoided in a further 26 (0.6%). Disease-specific treatment was started in 85 (2%) probands, including seizure-control medications and dietary supplements, and contra-indicated medications were stopped or avoided in a further 20 (0.5%). The option of prenatal/preimplantation genetic testing was discussed with 1204 (28%) families, despite the relatively advanced age of the parents at the time of diagnosis. Importantly, condition-specific information or literature was given to 3214 (76%) families, and 880 (21%) were involved in family support groups. In the most common condition (KBG syndrome; 79 [2%] probands), clinical interventions only partially reflected the temporal development of phenotypes, highlighting the importance of consensus management guidelines and patient support groups.

**Conclusion:**

Our results underscore the importance of achieving a clinico-molecular diagnosis to ensure timely onward referral of patients, enabling appropriate care and anticipatory surveillance, and for accessing relevant patient support groups.

## Introduction

Despite widespread use of genomic testing in children with developmental disorders (DD), relatively little has been documented about the outcomes after a genetic diagnosis in this group of patients.^1^ Steady advances in genomic technologies, including DNA microarray analysis and exome/genome sequencing, have resulted in the identification of a monogenic cause in around half of individuals affected with a presumed genetic DD.^2^, ^3^, ^4^ The most widely reported outcome from genome-wide sequencing is the diagnostic yield,^2^ but the clinical management implications of a diagnosis have been less well documented. The value of a diagnosis to the family may include genetic counseling, accessing patient support groups, and reproductive planning (https://www.undiagnosed.org.uk/support-information/what-does-getting-a-genetic-diagnosis-mean/).^5^ However, the value for clinical management has been less clearly documented, and it has sometimes been assumed that in many cases nothing different can be done to manage the affected child,^6^ rendering a precise molecular diagnosis an additional detail rather than a pivotal point in the ongoing management of the child and their family.

We sought to investigate outcomes in families affected by severe DD for which a genetic diagnosis was made through the Deciphering Developmental Disorders (DDD) Study, which recruited families affected by severe undiagnosed DD from across the United Kingdom and Republic of Ireland. Families were recruited, phenotyped, and managed by regional clinical genetics teams and were genotyped centrally to find novel genetic causes for their conditions. Likely genetic diagnoseswere communicated to clinicical teams via DECIPHER prior to discussion with families where relevant.^7^, ^8^, ^9^ By including outcomes data from across the whole of the United Kingdom and Republic of Ireland, we were able to systematically analyze interventions in >4200 diagnosed probands and evaluate the management of individuals affected by the same syndromes.

## Materials and Methods

### Eligibility

Probands with severe previously undiagnosed DD, as defined by the eligibility criteria,^10^ were recruited into the DDD study and analyzed using microarrays (array comparative genomic hybridization, 2X 1M probes, and Single Nucleotide Polymorphism [SNP] genotyping arrays) and exome sequencing, as described previously.^7^, ^8^, ^9^ Probands were selected for follow-up to investigate outcomes if they had received a likely diagnosis from the DDD study reported to referring clinical geneticists via DECIPHER^11^ as of 8 March 2021 (*n* = 5010), herein defined as a clinician-annotated pathogenic/likely pathogenic genotype,^4^ or de novo variant or biallelic loss-of-function variant in a curated database of known DD Gene-2-Phenotype (DDG2P) genes.^12^

### Data collection

Parental ages, quantitative growth data and Human Phenotype Ontology (HPO)^13^ terms were prospectively collected on all probands in the DDD study. A clinical outcomes questionnaire was subsequently designed based on a pilot study,^1^ including questions relating to treatment, testing/screening, reproductive choice, information provision, and adverse outcomes relating to receiving a diagnosis. In addition to single response questions (Table 1), further detailed information was collected in free-text format on specific medical interventions (treatments and testing/screening), referring specialties, and adverse outcomes (Supplemental Material). The questionnaire was codified into a standardized pro forma and circulated to each Regional Genetics Service to complete for their diagnosed DDD families using clinical notes from regional clinical genetics services, including a pseudonymized DECIPHER ID linked to the diagnosis for each proband. Data were collated from March 2021 to July 2022. Variants were confirmed in an National Health Service diagnostic laboratory where appropriate.

**Table 1:** Table 1 Summary of single response question results from 4237 diagnosed families in the DDD study

Topic	Question	Yes	No	Unknown
Interventions	Is a diagnosis-specific treatment available?^a^	144 (3%)	3928 (93%)	165 (4%)
Interventions	Were any one-off investigations performed as a result of diagnosis?^a^	749 (18%)	3325 (78%)	163 (4%)
Interventions	Was the proband referred to a different specialty for any screening?^a^	799 (19%)	3295 (78%)	143 (3%)
Interventions	Were there any interventions avoided as a result of diagnosis?^a^	53 (1%)	3884 (92%)	300 (7%)
Interventions	If diagnosis earlier could any interventions have been avoided?	418 (10%)	3387 (80%)	432 (10%)
Interventions	Additional research/clinical trials available?	1207 (28%)	2483 (59%)	547 (13%)
Reproductive	Has there been any pregnancies since the result?	235 (6%)	3006 (71%)	996 (24%)
Reproductive	Would PND be an option if family wished and applicable?	3029 (71%)	544 (13%)	664 (16%)
Reproductive	Was PND discussed in clinic?	1205 (28%)	2432 (57%)	600 (14%)
Reproductive	Was PND performed?	78 (2%)	3741 (88%)	418 (10%)
Reproductive	Was PGT discussed in clinic?	340 (8%)	3293 (78%)	604 (14%)
Reproductive	Was PGT performed?	31 (1%)	3809 (90%)	397 (9%)
Information/support	Is diagnosis-specific information available?	2798 (66%)	1076 (25%)	363 (9%)
Information/support	Was this information given/signposted to the family?	2480 (59%)	1308 (31%)	449 (11%)
Information/support	Were the family given any scientific literature about the condition?	1563 (37%)	2189 (52%)	485 (11%)
Information/support	Were the family included in a scientific article?	772 (18%)	2651 (63%)	814 (19%)
Information/support	Has the family been involved in a patient support group?	880 (21%)	1395 (33%)	1962 (46%)
Adverse outcomes	Are there any known adverse outcomes?^a^	95 (2%)	3750 (89%)	392 (9%)

*DDD,* Deciphering Developmental Disorders; *PGT*, preimplantation genetic testing; *PND*, prenatal diagnosis.

a Further information requested in free-text form in separate table (see Supplemental Material).

## Results

### Overview of cohort

**Figure 1:**
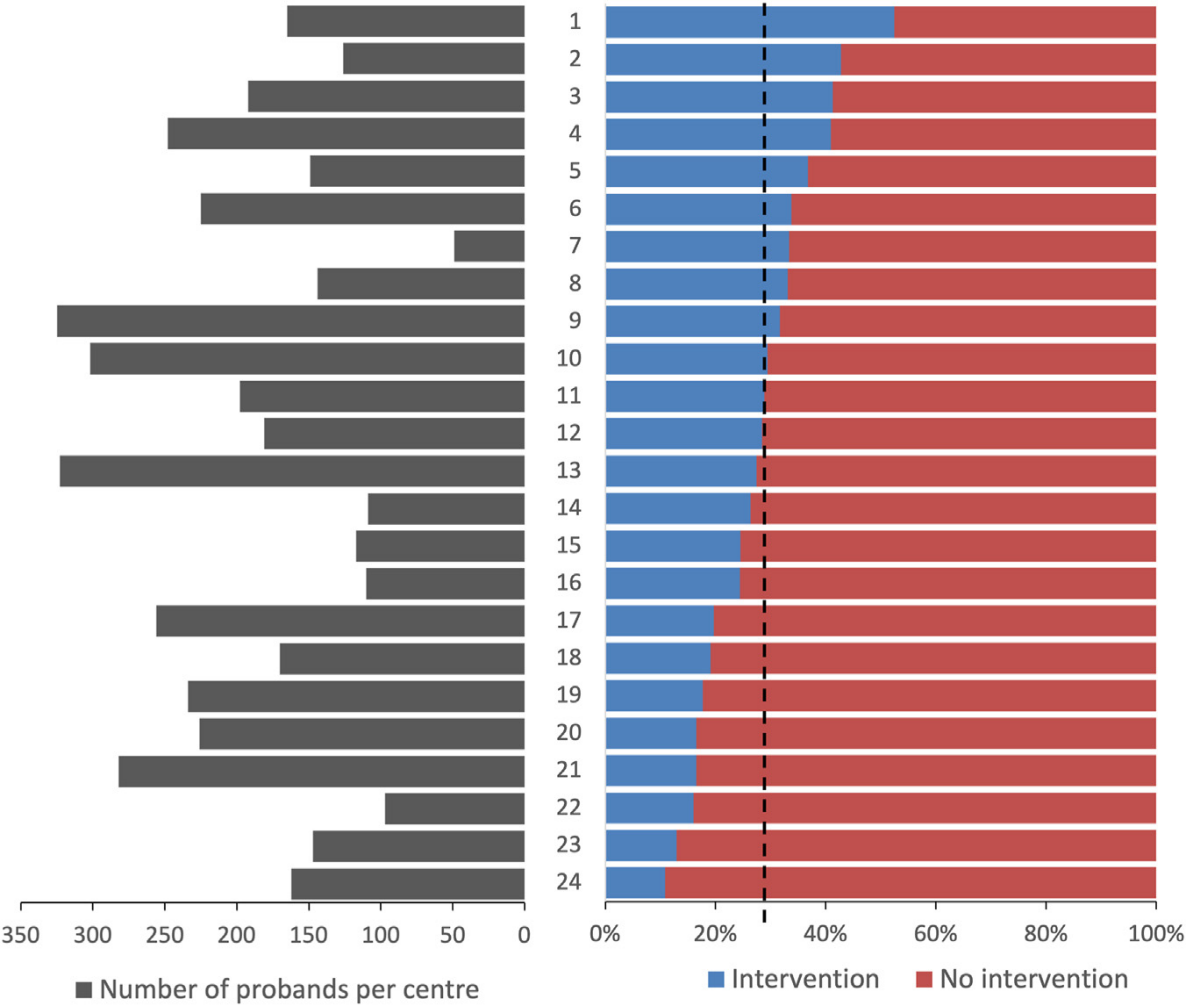
**Summary of diagnosed DDD probands per center.** Number of diagnosed Deciphering Developmental Disorders probands included in study (left) and percentage with interventions (treatment or testing; right) separated by the 24 Regional Genetic Services across the UK and Ireland. Black dotted line = mean across study.

**Figure 2:**
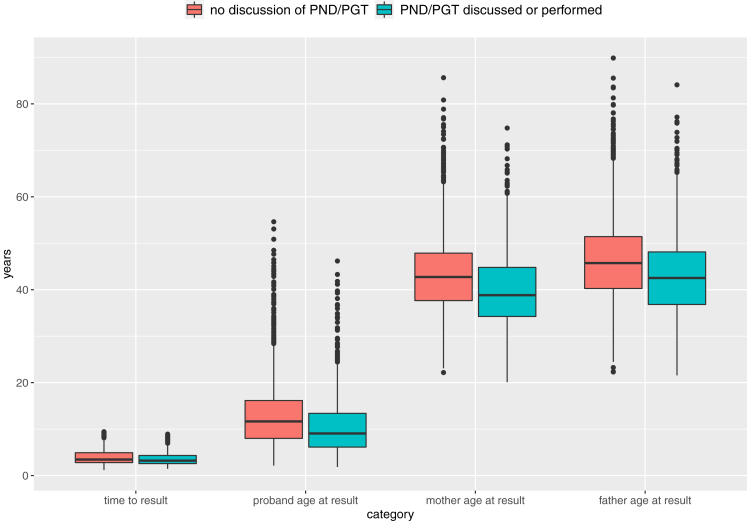
**Time to result and age of probands and parents at the point of diagnosis.** Green, prenatal testing discussed or performed; PGT, preimplantation genetic testing; PND, prenatal diagnosis; red, no record of prenatal testing being discussed with the family.

Outcomes data were recorded on 4237 diagnosed DDD probands (47% female) by 24 Regional Genetics Services across the United Kingdom and Republic of Ireland (range = 42-316 probands per center, Figure 1). Diagnoses spanned >800 unique rare conditions and included both small single-gene variants and large multigenic structural variants, with inheritance patterns including autosomal dominant (68% de novo, 7% inherited from an affected parent, and 8% with unknown inheritance), autosomal recessive (11%), X-linked (5%), and multiple diagnoses with different inheritance classes (1%).^4^ The median time from recruitment to result was 3.4 years (range: 1.1-9.4 years), at which point probands were a median of 11 years old (range: 1.8-55 years) and parents were a median of 43 years old (range: 20-90 years; Figure 2).

### Management of proband

Clinical outcomes that occurred following a diagnosis in 4237 DDD families are summarized in Table 1. Importantly, clinical management of the affected individual was reported to have changed in 28% (*n* = 1183) of diagnosed DDD probands as a result of receiving a genetic diagnosis, which ranged from 11% to 52% across the different regional genetics services (Figure 1). Clinical management is here defined to mean any treatment, testing, or screening of the proband, which could have been started, stopped, avoided, or reviewed; it excludes prenatal testing because this does not relate directly to management of the proband’s health, and joining support groups or accessing special educational services such as these are not directly clinical. This range may reflect differences in workforce capacity across different centers. There were no differences in rates of interventions between male and female probands, and no major differences between genetically defined ancestry groups (albeit within a cohort with limited diversity).^4^

**Figure 3:**
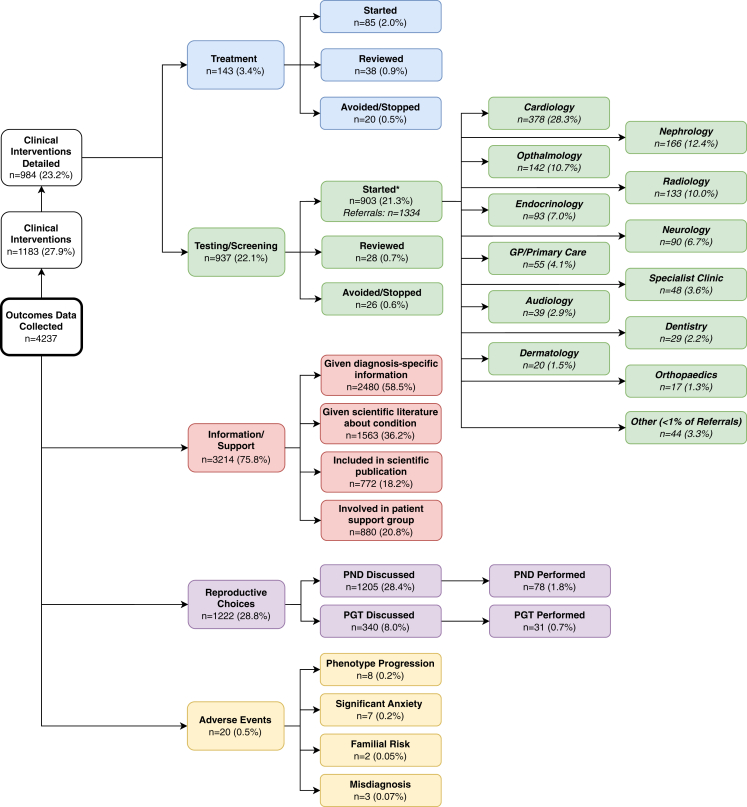
**Flowchart summarizing individual-level outcomes following a genetic diagnosis in the DDD study.** Richer data collected using a mixture of controlled vocabulary and free text (see Supplemental Material). Individuals who experienced more than 1 outcome are included multiple times (ie, in all relevant boxes). ∗Some probands had >1 test initiated as a result of their diagnosis. PGT, preimplantation genetic test; PND, prenatal diagnosis.

Detailed individual-level information about specific interventions was available for the majority of those in whom clinical management was altered (83%; *n* = 984) and is summarized in Figure 3. Treatment was altered in 143 probands (3%), which included starting, reviewing, stopping, or avoiding specific therapies. Recurrently prescribed medications included drugs to control seizures (eg, carbamazepine, clonazepam, lamotrigine, and topiramate) and specific dietary supplements (eg, folate, creatinine, carnitine, and ornithine). Interventions include probands who accessed prophylactic treatment to reduce the risk of condition-specific complications (eg, retinal detachment in Stickler syndrome). Further medical investigations were performed in 937 probands (22%) through referral to a wide range of nongenetics specialists for further clinical input to manage associated phenotypes, including screening and/or nongenetic diagnostic testing. The largest number of referrals were made to cardiology (28%), followed by nephrology (13%), ophthalmology (11%), radiology (10%), neurology/pediatric neurology (7%), endocrinology (7%), primary care (4%), condition-specific or specialist metabolic clinics (4%), audiology (3%), dentistry (2%), dermatology (2%), orthopedics (1%), and ear, nose, and throat, respiratory, general pediatrics, psychiatry/clinical psychology, and urology (all <1%). A third of referred probands were referred for multiple different investigations or to multiple nongenetics specialists to manage different aspects of their phenotype, highlighting the complexity of genetic DD syndromes. Free-text information gathered also indicated that additional phenotypic features were detected and managed in many probands after these referrals, reflecting the value of timely diagnosis and referral for identifying complications and providing appropriate multi-disciplinary care. In 418 probands (10%), it was reported that some interventions (such as magnetic resonance imaging scans and muscle biopsies) could have been avoided if the diagnosis had been made earlier.

### Management of family

In addition to medical management of the affected proband, we also investigated wider clinical management of the family following their diagnosis. Condition-specific information or support was provided to 3214 families (76%), including scientific literature and/or patient information leaflets. Remarkably, 772 (18%) of families had been included in condition-specific scientific publications, which likely reflects the rarity and recent discovery of many of the disease-associated genes. At the time of data collection, prenatal diagnosis or preimplantation genetic testing had been discussed with 1222 families (29%) and performed in 103 (2%). It is likely these proportions would have been higher had parents been younger at the point of receiving the diagnosis (Figure 2), and only 235 (6%) of parents had a confirmed pregnancy since receiving their child’s genetic diagnosis. Finally, reflecting the fact that receiving a diagnosis does not always provide welcome news, a diagnosis-related adverse outcome was reported in 20 families (0.47%), in whom parental or patient anxiety resulted in additional clinic appointments. Reasons given for anxieties related to a range of issues, including the possibility of phenotype progression (based on other individuals affected with the same condition), the prospect of additional interventions, the lack of diagnosis-specific information, potential risks to other family members, and changes to a previous diagnostic result (either a previous missed or misdiagnosis^14^).

### Data aggregation to build knowledge

**Figure 4:**
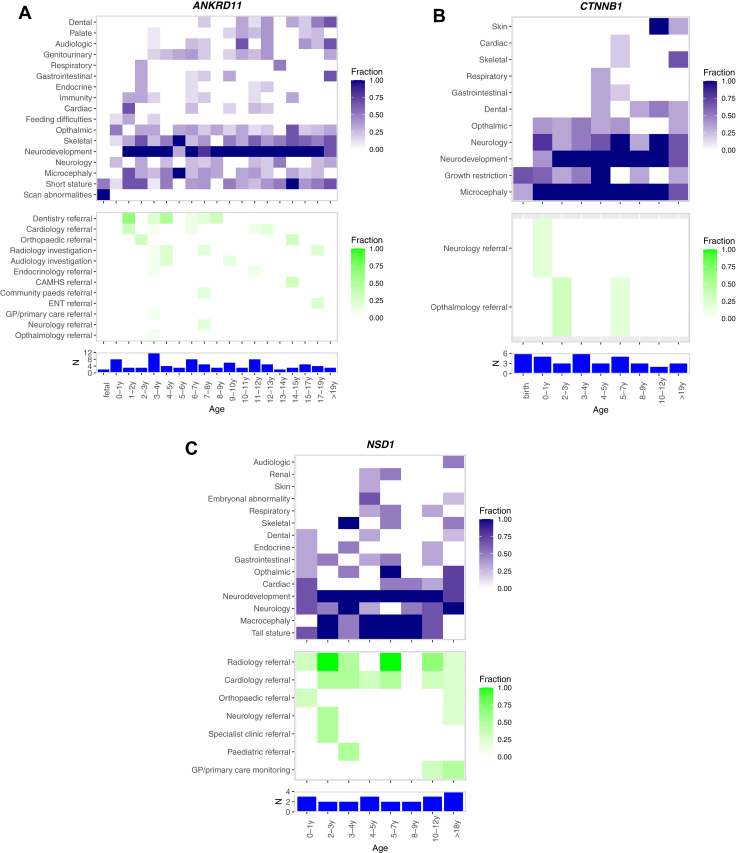
**Quasi-natural history of disease and summary of interventions for DDD probands diagnosed with the same condition.** A-C. (A) *ANKRD11,* (B) *CTNNB1*, and (C) *NSD1.* Top panel—heatmap of phenotypes grouped by system and age; middle panel—heatmap of interventions grouped by age; bottom panel—histogram of number of probands in each group based on age at recruitment.

We further sought to compare phenotypes and outcomes between probands of different ages diagnosed with the same condition. In our data set, 37 genes had diagnostic variants in >20 probands, together accounting for 1218 (29%) of diagnoses.^4^ Of these, we focused on 3 well-established exemplar genes: *ANKRD11* (KBG syndrome; *n* = 79),^15^ which has the largest number of DDD diagnoses; *CTNNB1* (neurodevelopmental disorder with spastic diplegia and visual defects; *n* = 30),^16^ in which there is a clinical imperative for ophthalmic surveillance; and *NSD1* (Sotos syndrome; *n* = 20),^17^ in which the highest proportion of DDD probands (65%) had medical interventions after a diagnosis. Using HPO terms and quantitative phenotypes grouped by age and system, we created a quasi-natural history for the conditions and overlaid information about when and how often particular interventions occurred (Figure 4).

For *ANKRD11,* the phenotype heatmap (Figure 4A) demonstrates a multisystem disorder with variable expression. Short stature and neurodevelopmental features are strongly consistent throughout the age range, but there is an age-dependent emergence to other features, such as dental and audiologic phenotypes. The spread of other phenotypes is consistent with the body of literature already available in KBG syndrome but demonstrates a highly visual quasi-natural history, useful for both parents and clinicians alike when determining management plans at a point in time. Interventions in *ANKRD11* patients demonstrated large-scale variability across the group, which is likely associated with the timing of the emergence of published clinical recommendations.^18^ In contrast, the heatmap for *CTNNB1* (Figure 4B) illustrates a more tightly defined range of phenotypic features, demonstrating a severe early onset neurodevelopmental disorder with postnatal onset microcephaly. Interestingly, we did not observe a consistent pattern of ophthalmology referrals among these patients, despite a 40% risk of retinal detachment requiring regular eye surveillance to prevent total blindness.^19^ This observation is potentially due to variability in data collection for onward referral and the severity of the phenotype precluding referral but suggests an opportunity to alert clinicians to the need for ophthalmology referral in these patients. By comparison*,* the well-documented recommendations for baseline investigations and referrals were evident in our data for in *NSD1* (Figure 4C), as was the established evolution of the phenotype with age.^20^ Interestingly, although patterns of phenotype progression are apparent with increasing age, all 3 conditions show a degree of variable expressivity, with only a few phenotypes universally present. Clinical interventions across all 3 conditions appear to be somewhat sporadic and only partially reflect the temporal development of phenotypes, suggesting that systematic improvements could be made to referral practices to ensure equity of access to the most appropriate care.

### Benefits of support groups

Finally, we found that 880 (20.8%) of diagnosed DDD families were involved in patient support groups. In addition to umbrella patient organizations supporting families with genetic conditions and pre-existing condition-specific organizations, numerous new condition-specific patient support groups were created as a direct result of disease-gene discovery in the DDD study. These groups range from small parent-led social media (eg, Facebook) groups, that bring patients and families together to share experiences, to the development of registered charities and foundations. We also note that, over the course of the study, DDD clinical collaborators have contributed to authoring >40 single-gene patient information leaflets in collaboration with Unique (https://rarechromo.org/disorder-guides/).

## Discussion

We have retrospectively recorded and analyzed outcomes after a genetic diagnosis in 4237 families in the DDD study. We have shown that around a quarter of individuals affected by a severe DD received a change in medical management after their genetic diagnosis, primarily through a range of referrals to nongenetics specialties for additional testing and surveillance. The clinical impact of a precise molecular diagnosis on the management pathway for an individual patient thus enables a precision medicine approach and the provision of appropriate care, sometimes preventing particular phenotypes from developing. The likely increased demand for specialist assessments following a genetic diagnosis also needs to be costed and provided. Additionally, at least three-quarters of families were given condition-specific information, which supports understanding and family adaption to a genetic diagnosis. Very few adverse outcomes were reported, suggesting that the anxiety and other mental health implications associated with receiving genetic results from a large genomics research study delivered via an expert clinical service were generally low.

We have also presented a novel approach to displaying a quasi-natural history of specific genetic conditions, using data from multiple affected probands of different ages. The richness of phenotype data in KBG syndrome in particular shows the variable expressivity of this highly penetrant condition and highlights when and how likely particular phenotypes are to manifest. However, the link between the emergence of clinical phenotypes and the necessary clinical interventions is weak and may vary both within a condition and between services. This may be due to the necessary inclusion of data from multiple different individuals, often with different causal variants (albeit within the same gene and with the same predicted effect); therefore, the differences may not wholly reflect phenotypic progression. Nonetheless, we hope that these representations of phenotypes and intervention data with age will provide better prognostic information to clinicians and patients and catalyze the development of consensus management guidelines. In addition, the growing size and number of disorder-specific family support groups should be recognized and welcomed by both the clinical and patient communities and may provide a mechanism by which referral and clinical management practices could be compared and optimized. Support groups play a vital role in the provision of information and act as a forum for patients and families to share experiences and seek advice from people in a similar situation.^21^ Parents and carers of children with DD are at risk of social isolation and emotional distress, which can be exacerbated when the condition is rare.^22^^,^^23^ Many participants of support groups report positive outcomes, such as reduced isolation and anxiety, improvements in coping skills and increased self-esteem and empowerment.^24^ Internet-based support groups also mean that geographical location is no barrier to accessing support and making connections with others.^25^ Ultimately, bringing together patients, clinicians and researchers with a common focus on a specific condition can stimulate research, enabling codevelopment of research questions and providing a vehicle for both recruitment and dissemination of findings.

This large-scale nation-wide study was made possible through an extensive network of regional clinical collaborators across the National Health Service in the United Kingdom and the Health Service Execuive in Ireland. However, there are significant challenges to gathering comparable data on thousands of families under the care of hundreds of clinicians spread across 24 different sites. Because of the large size and geographical spread of the study, we did not attempt to gather information directly from parents or probands relating to social, educational, or other nonclinical outcomes, although there is little doubt that receiving a formal diagnosis can be of immense value to families. Provision of social, financial, and educational support should be based on an individual’s need, but families often report that a diagnostic label can be extremely helpful when advocating for their child’s needs.^1^^,^^26^^,^^27^ Within each clinic, individual data collectors were limited to information available in their local genetics notes, in which the level of detail routinely recorded can vary substantially—exacerbated by the move from paper toward electronic health records—hampering our ability to compare findings between services. Moreover, the size and expertise of data collection teams varied across the sites, potentially resulting in different ways of reporting similar outcomes. There may also be differences between clinicians and regions in referral practices (eg, refer versus test onsite), as well as the timing and purpose of testing (diagnostic versus screening, etc).

We were also limited by the retrospective collection of outcomes data, recorded at a single point in time but relating to diagnoses returned over the course of a 7-year period. This approach cannot account for the development of clinical guidelines and dissemination of best practice over time. This issue is exemplified by KBG syndrome, for which clinical management recommendations were published in 2016, after most *ANKRD11* diagnoses were returned in DDD.^18^ Similarly, we were limited by the collection of phenotypes at recruitment, which does not take account of phenotypic progression. It was not always possible to determine whether a particular clinical action resulted directly from the genetic diagnosis or from the appearance of a phenotype. Our results are skewed both by the high proportion of diagnostic de novo variants and the relatively advanced age of parents at the point of receiving a diagnosis, which may have reduced the appropriateness of reproductive counseling and limited parental opportunity for further testing. Finally, even within our large data set, because of the rareness of individual conditions (with >800 different rare diseases diagnosed to date within this cohort), there were relatively small numbers of probands with the same conditions, which reduced our ability to create accurate quasi-natural histories across different age groups. Ideally, longitudinal phenotype collection on individuals would enable true natural histories to be collected and compared, and the aggregation of data on larger numbers of patients through databases such as DECIPHER will enable these data to be systematically analyzed and widely shared.

In conclusion, we have demonstrated that it is both possible and useful to collect outcomes data from clinical genetics services on the impact of receiving a genetic diagnosis. Making an accurate genetic diagnosis is often crucial for directing clinical management of affected individuals and providing advice regarding risks to other family members, including reproductive advice. Although molecularly targeted treatments for monogenic DDs are still limited, more will no doubt become available as new technologies develop. Our findings highlight the importance of onward referral to ensure the best care for patients and families affected by rare diseases and also underscore the value of developing best practice guidelines to ensure equity of access to appropriate clinical interventions.

## Data Availability

Sequence and variant-level data and phenotypic data for the Deciphering Developmental Disorders study data are available from the European Genome-phenome Archive (EGA; https://www.ebi.ac.uk/ega/) with study ID EGAS00001000775. Clinically interpreted variants and associated phenotypes from the Deciphering Developmental Disorders study are available through DECIPHER (https://www.deciphergenomics.org/).

## Conflict of Interest

The authors declare no conflicts of interest.

## Additional Information

Supplemental Material Pro forma tables for collecting outcomes data.
